# “In the end, the story of climate change was one of hope and redemption”: ChatGPT’s narrative on global warming

**DOI:** 10.1007/s13280-024-01997-7

**Published:** 2024-03-02

**Authors:** Bernd Sommer, Sarah von Querfurth

**Affiliations:** 1 Environmental Sociology and Transformation Research, TU Dortmund University, Dortmund, Germany; 2 Department of Social Sciences, TU Dortmund University, Emil-Figge-Str. 50, 44221 Dortmund, Germany

**Keywords:** Artificial intelligence (AI), ChatGPT, Climate change, Climate change narratives, Climate justice, De-politicization

## Abstract

AI chatbots such as ChatGPT help people produce texts. According to media reporting, these texts are also used for educational purposes. Thus, AI influences people’s knowledge and perception of current issues. This paper examines the narrative of ChatGPT's stories on climate change. Our explorative analysis reveals that ChatGPT’s stories on climate change show a relatively uniform structure and similar content. Generally, the narrative is in line with scientific knowledge on climate change; the stories convey no significant misinformation. However, specific topics in current debates on global warming are conspicuously missing. According to the ChatGPT narrative, humans as a species are responsible for climate change and specific economic activities or actors associated with carbon emissions play no role. Analogously, the social structuration of vulnerability to climate impacts and issues of climate justice are hardly addressed. ChatGPT’s narrative consists of de-politicized stories that are highly optimistic about technological progress.

**Supplementary Information:**

The online version contains supplementary material available at 10.1007/s13280-024-01997-7.

## Introduction

The development of artificial intelligence (AI) and especially natural language processing (NLP) is changing the way that information is processed in society. ChatGPT—short for Chat Generative Pre-trained Transformer—is particularly popular as it is able to generate structured texts of various genres that are hard to distinguish from texts written by humans. ChatGPT offers a zero-priced version of an AI chatbot and is easy to access. According to media reporting, many people already use AI chatbots to produce texts they need for lectures, homework, presentations or similar purposes (Rudolph et al. 2023). Therefore, such developments might have a significant impact on how people discuss socially relevant topics such as anthropogenic climate change.

ChatGPT is a software application that uses artificial intelligence to produce texts by grabbing data from the internet. ChatGPT’s “knowledge” mirrors the information it was trained on up to September 2021 (Azaria 2022). However, it is unclear which information was chosen. Despite the scientific consensus that global warming is human-made, much mis- and deliberate disinformation on contemporary climate change exists in the internet (Treen et al. 2020). On the one hand, this is because of a well-financed denial countermovement that challenges scientific knowledge on climate change via organizations and publications in various media (Oreskes and Conway 2011; Dunlap and McCright 2015). On the other hand, due to the journalistic norm of balanced reporting, prestige media tends to relativize the scientific consensus on climate change by drawing inappropriately often on marginal views on the causes for climate change as well as mitigation issues (Boykoff and Boykoff 2004). Furthermore, there are diverging views within societies on the actions that should be taken to tackle the climate crisis and achieve ecological sustainability (Adloff and Neckel 2019). Finally, the framing of the topic shapes opinions on and the perception of global warming. These different storylines lead to different approaches to solutions for climate change (Nisbet 2014). For these reasons, the kind of climate change story open AI software such as ChatGPT (re)produces is highly relevant.

So far, there is very little research on the climate-related content produced by AI chatbots (Bergener et al. 2023). A study by Zhu et al. (2023, p. 17,668), not dealing with climate change but with current topics in environmental research such as per- and polyfluoroalkyl substances (PFAS), found that ChatGPT is “able to provide useful general information,” but fails to provide correct detailed knowledge. Given this research gap, this paper systematically examines which narrative ChatGPT generates when writing stories on climate change. After introducing the research design and methodology, the key findings of our analysis are presented by giving an overview on the general structure and content of ChatGPT’s stories on climate change. In a second step, we interpret our findings in the light of the academic literature on climate change, its causes and impacts as well as their respective societal dimensions. By doing so, it becomes apparent which themes and aspects are represented in ChatGPT’s climate change narrative and which are not. We draw on a conceptual framework suggested by Adloff and Neckel (2019) that distinguishes three “futures of sustainability”: modernization, transformation, and control. The framework enables us to identify diverging trajectories of social change based on practices, imaginaries as well as notions relating to social structure, i.e., power relations in a society. We conclude the paper with a discussion of the limitations of our analysis as well as suggestions for further research.

## Materials and methods

We obtained our data by asking OpenAI’s ChatGPT to write a story on climate change. The prompt we chose was: “Write a story on climate change.” We rejected alternative expressions such as “climate crisis”—which is used by some newspapers as well as activists—because the term “climate change” is more commonly used in official and academic sources. Furthermore, we tried to use a relatively generic prompt that requires no specific prior knowledge and is therefore closer to what is likely to be used by non-specialists and ordinary users. In other words, we did not address specific aspects of the topic, such as “climate change mitigation,” “climate change adaptation,” and “climate impacts.” The idea was to analyze ChatGPT’s general narrative on climate change.

**Fig. 1:**
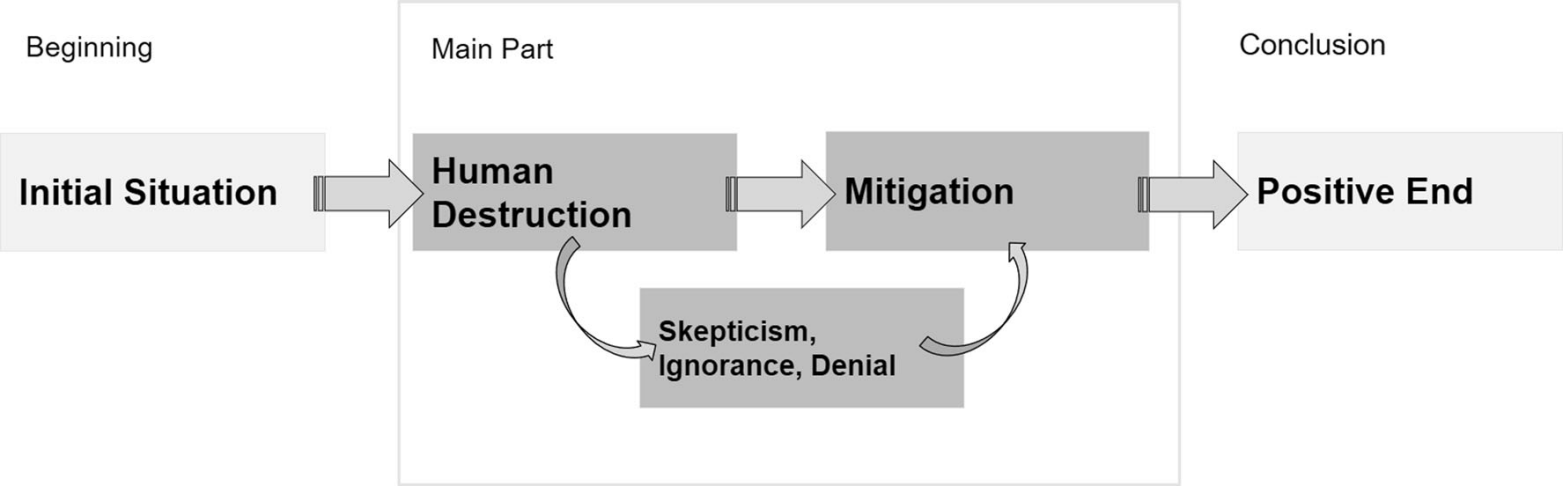
Storyline of ChatGPT’s stories on climate change

The free version of ChatGPT allows around 380 words per story. We applied a theoretical sampling approach as used in Grounded Theory (Strauss and Corbin 1990), meaning that we analyzed and acquired data in a circular process. Initially, we analyzed the text material sequentially and applied open coding to generate inductive codes. We constantly went back and forth in the material and compared the stories, adding new codes. We generated new material in ChatGPT by clicking “regenerate response” until the stories contained no new content matter that could not be covered by hitherto generated codes. Generally, the quality of qualitative social research is not directly related to the size of the database but follows the principle of saturation which is reached “when the collection of new data does not shed any further light on the issue under investigation” (Mason 2010). After 13 repetitions, we reached “theoretical saturation” (Krotz 2018, p. 63), meaning that ChatGPT delivered 14 different stories on climate change. We generated the stories between March 3 and March 5, 2023, with the free version of ChatGPT 3.5. These stories contain 5279 words in total. Together they form the database for our first analysis (Fig. 1).

**Fig. 2:**
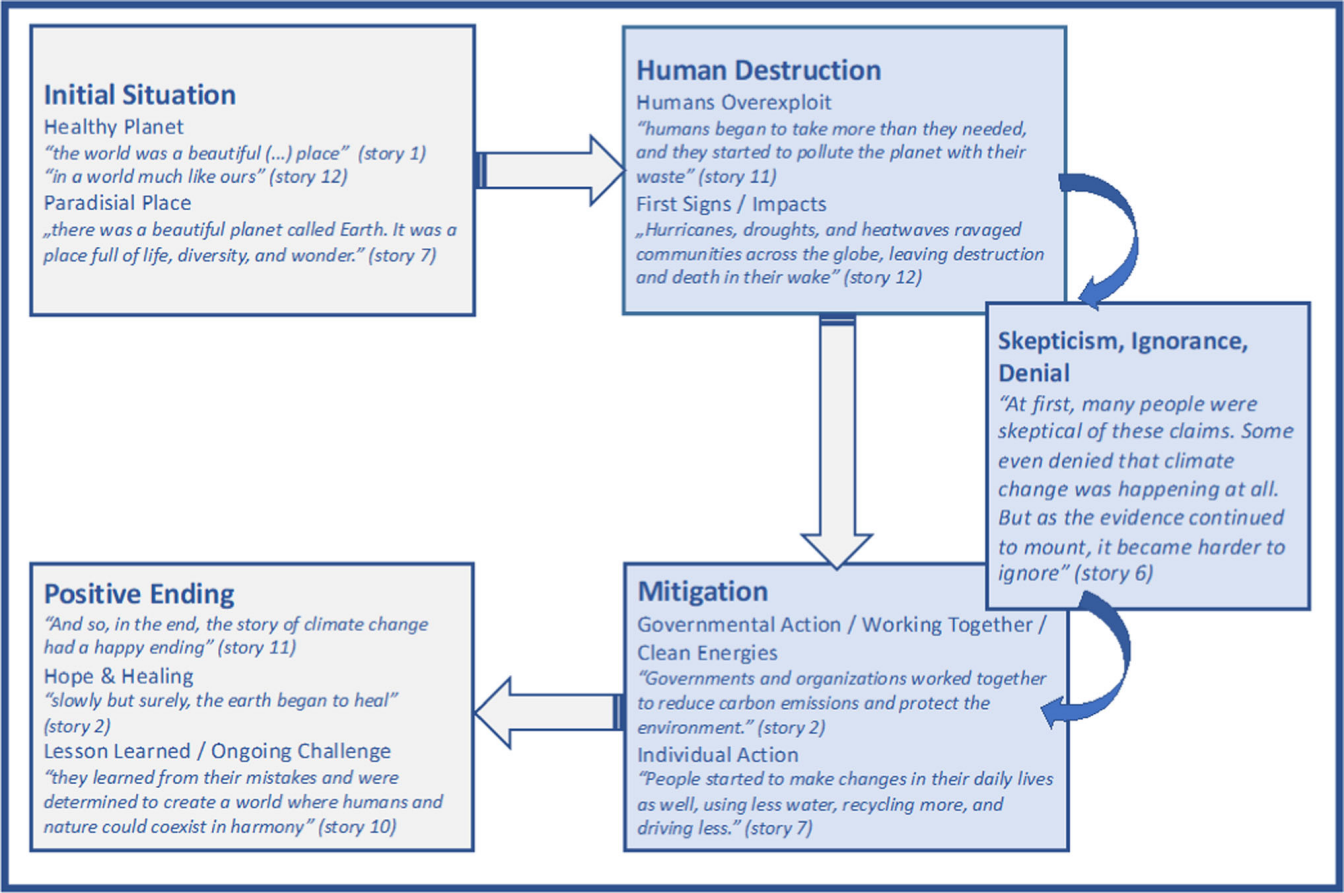
Schema of ChatGPT’s stories on climate change based on the storyline matrix. Quotes are typical examples from various stories

Open coding helped us to subdivide and initially structure the material. The codes followed the material and were often constructed in vivo, meaning that they are literal passages from the data. We coded the structure and wording of the material by transforming the empirical data (sequences in the stories) into more abstract concepts. In a next step, we coded the material axially, creating new categories that integrated various concepts which are informed by climate research such as “mitigation,” “extreme weather” or “cryo- and hydrosphere.” We subsequently used these key categories for selective coding of the complete material (see Appendix S1). Additionally, we drafted a matrix for every story that summarizes its individual storyline (see Appendix S2). This was the basis for developing a schema for ChatGPT’s stories on climate change, which shows how the various key categories are related (see Fig. 2).

Grounded Theory Methodology (GTM) does not clearly separate the sampling phase from the analysis phase of the research process. GTM encourages resampling by collecting material according to the principals of minimal or maximal contrast (Przyborski and Wohlrab-Sahr 2014). Based on our finding that the issue of climate justice is hardly addressed in the initial 14 stories, our resampling consisted in generating new stories with the prompt “Tell us a story about climate justice.” These new stories were also generated by the free version of ChatGPT 3.5, but at a later date: November 2023. First, we again used the initial prompt (“Write a story about climate change”), in order to make sure that the narrative has not changed in a meaningful way between March and November 2023. Afterward, five more stories were generated with the prompt (with a total of 2594 words). These stories were included in the second phase of our analysis.

We used memos throughout the whole research process to capture our thoughts on the material. To analyze the data, we used the software MAXQDA, which has been specifically developed for computer-assisted qualitative data analysis.

## Results

### General structure and content of ChatGPT’s stories on climate change

On the whole, all stories generated by ChatGPT featured an analogous structure and contained similar content which we present here. While the term “story” refers to both a narration of fictional as well as non-fictional (“real”) events, each of ChatGPT’s stories is fictional and begins with the words *“Once upon a time”*. However, the generated narratives are not entirely fictitious. Every story deals with the anthropogenic global warming of our time, even though our initiating stimulus contained no epochal specification and “climate change” is a process that has occurred many times in earth’s history due to natural factors of our planet (Masson-Delmotte et al. 2021).

All stories include a short introduction, a main section and usually an ending that presents a moral sentiment. The main characters are humans; time and place are usually not mentioned. Thus, the stories exhibit features of a parable. The brief introduction includes a description of the initial situation in which the planet is a *“beautiful”* place (e.g. story 1) or, alternatively, *“similar to our world”* (e.g. story 4). The essence of all introductory sentences is that this planet assures the livelihood of diverse species: *“there was a planet that was teeming with life. There were lush forests, sparkling oceans, and vast expanses of grasslands where all sorts of creatures roamed free”* (story 4).

Some stories almost seem to resemble the creation story from the Pentateuch 1 Moses; Genesis 1:1–3:24 (The Holy Bible 2008). The earth is described as being similar to the scriptural Garden of Eden. Most stories then proceed to a narration about humans exploiting this paradisial place, which leads to climate change, or the expulsion from Paradise, that not only affects humanity today but also the following generations. In story 2, this parallel is especially manifest:

Once upon a time, the world was a beautiful and pristine place. The air was fresh, the water was clean, and the forests were lush and green. But as time went on, humans began to use the earth's resources without regard for the consequences. They burned fossil fuels, cut down forests, and polluted the air and water. And soon, the effects of their actions began to show.

The stories repeatedly highlight humans’ responsibility for climate change by referring to scientific evidence (e.g. story 3). The causes of climate change in the stories are carbon emissions, the use of fossil fuels, deforestation, waste, destroyed ecosystems, and overconsumption. Not all causes are mentioned in every story, but all mention carbon emissions as a reason. One story (5) contains a peculiar variation: Here, the story takes place in a village, and information on the causes of the changing climate are provided by a *“wise man”*.

The first signs of climate change are *“subtle”* (e.g. story 13) and include changes in temperature and weather as well as impacts on the hydro- and cryosphere. In many stories, endeavors to ignore climate change, being skeptical about it, denying it, or making efforts to adapt to climate change without measures for mitigation are mentioned. However, these attempts proved to be impossible as the life of people around the world becomes more and more threatened by disasters and extreme weather events, such as heat waves, fires, hurricanes, floods, droughts, and the extinction of species.

The stories continue that these threats convince many people, governments, organizations, and companies to engage in climate mitigation. A driver for change is activism: *“concerned citizens joined forces to raise awareness about the dangers of climate change”* (story 12) and they *“organized protests and rallies, wrote letters to their elected officials, and started a social media campaign to raise awareness about the urgency of the climate crisis”* (story 8). Additionally, individual lifestyle changes to reduce personal carbon footprints by driving less, reducing water consumption, and by avoiding waste are mentioned. In story 7, all individual changes that are addressed by ChatGPT in the stories come together: *“People started to make changes in their daily lives as well, using less water, recycling more, and driving less”*. Sometimes, lifestyle changes are addressed rather vaguely, as in story 14 *“many began to adopt more sustainable practices in their daily lives”* and in story *13 “people began to make changes in their own lives”*.

Every story highlights the importance of cooperation. For example, story 2 states: “*And that by working together and taking action, we can protect the earth and ensure a bright future for ourselves and for generations to come”*. Story 1 uses an even stronger wording, calling the fight against climate change *“a testament to the power of unity”*. Suggested policies mentioned for mitigating climate change are reducing the burning of fossil fuels by investing in or using renewable energies, investment in clean technology afforestation, and a more sustainable economy (e.g. story 12). In story 13, general technological progress helped to mitigate climate change.

The end of all stories, except one, is positive: Due to human action, the planet *“heals”* (e.g. story 1) and becomes a better place again. Story 3 summarizes: *“In the end, the story of climate change was one of hope and redemption”*. All stories except one have the aspect of a moral sentiment. The *“lesson learned”* (e.g. story 5) is that climate change mitigation requires *“hard work”* (story 4) but is essential to maintain the planet as a livable place. All stories frame climate change as an ongoing *“challenge”* (story 14) in which *“they [the people] had the power to make a difference”* (story 5). One story had an open ending, as people *“refuse to take action”* (story 6). In this story, *“the planet continued to warm”* but there is still hope for the planet, as some people *“fought to make a difference”* (story 6). The structure and content of ChatGPT’s stories on climate change are schematically visualized in Fig. 2 (for a detailed overview of the topics and themes addressed see Appendix S1).

Changing the prompt to “Tell us a story about climate justice” does not alter the general structure of the narrative. Only certain passages and formulations change, in particular addressing the aspect that climate change impacts vulnerable communities more severely. For instance, the protagonists of story 16 “*realized that climate change was not just an environmental issue; it was a matter of justice, with the most vulnerable paying the highest price”*. For a detailed overview of ChatGPT’s stories on climate justice see Appendix S3.

## Discussion

### Crisis, ecological modernization, and faulty humans without society

Although we did not use the term “climate crisis,” ChatGPT’s stories on climate change exhibit the features of a crisis narrative: At the beginning, humans and nature reside on a flourishing planet. Due to the overexploitation of natural resources and the burning of fossil fuels, this state is shattered and climate change impacts disastrously on societies and nature. Governmental interventions, sometimes triggered by activism, lead to the introduction of new technologies that reduce emissions, and, finally, the pre-crisis state can almost be reestablished—though climate change remains an *“ongoing challenge”* (story 14).

In the context of sustainability, Adloff and Neckel (2019) identify three ideal typical trajectories of social change: modernization, transformation, and control. “Ecological modernization” can be characterized as an approach that is highly optimistic about technological progress and affirms the foundations of today’s political economy (Adloff and Neckel 2019). “Transformation,” in contrast, includes support for fundamental social change as well as questioning the imperative of economic growth (Adloff and Neckel 2019). Finally, “control” refers to perspectives that try “to solve the problems of sustainable development with wide-ranging politics of control, using concepts such as ‘ecological state of emergency’ or enforcing resilience measures for vulnerable populations while creating safe enclaves for a privileged few” (Adloff and Neckel 2019, p. 1015). According to Adloff and Neckel (2019, p. 1015), “these imaginaries then structure distinctive practices of sustainability in the fields of politics, the economy, civil society, and science.” Adloff and Neckel’s (2019) conceptual framework can serve as a useful heuristic for discussing ChatGPT’s stories of climate change.

ChatGPT’s stories follow the notion of “ecological modernization.” Ecological modernization programs mainly promote environmental improvements through technological innovations. According to this concept, clean or green technologies—such as renewable energies—spread via markets due to a favorable regulatory framework (Jänicke 2008). Story 8 expresses this idea succinctly: *“Governments around the world began to take action, implementing policies to reduce greenhouse gas emissions and transition to renewable energy sources.”* On the other hand, ecological modernization programs “do not intend to fundamentally alter existing *structures*—such as liberal democracy and market capitalism […]” (Adloff and Neckel 2019, p. 1018). In keeping with this notion, none of ChatGPT’s stories contains the imagery of transformation, i.e. more radical approaches such as *degrowth* that are discussed by social movements as well as in academia in response to the climate crisis (D’Alisa et al. 2015; Jackson 2017; Hickel 2021; Schmelzer et al. 2022). Different forms of political activism are mentioned occasionally in the stories (e.g. story 8), but different options and alternatives for dealing with the climate crisis are not presented in any of the stories.

Nor does the trajectory of control appear in ChatGPT’s stories of climate change. This only changes when altering our prompt to “Tell us a story about climate justice.” The ensuing stories do contain some control imagery: Story 18 elaborates that “the wealthy elite of Verdentia seemed shielded from these effects, living in climate-controlled luxury”—which is very close to Adloff and Neckel’s creation of “safe enclaves for a privileged few” (Adloff and Neckel 2019, p. 1015).

Theories of liberal as well as radical democracy view societal conflicts between divergent interests and perspectives as productive forces for social change (Dahrendorf 2012) or regard antagonistic positions as the core of the political (Mouffe 2005). Vice versa, if *there is no alternative* (infamously known as the TINA principle), political discourses become suspended. Writing on climate change policies, Machin (2013) has shown that consensus is not only an illusion, but democratic differences about the best policies are also necessary for collective action. The focus on technology and the lack of alternative approaches fits to ChatGPT’s description of the genesis of the climate crisis. While ChatGPT’s stories do repeatedly refer to specific societal actors in tackling the climate crisis, namely activists (e.g. story 12), companies (e.g. story 12), governments (e.g. story 2), NGOs (story 2), and scientists (e.g. story 3), this differs distinctly from the sequences dealing with the causes of climate change. Climate change is caused by *“human activity”* (e.g. story 3), simply *“humans”* (e.g. story 2), or *“the people of the world”* (e.g. story 1). Neither are specific societal actors identified, nor are the societal or economic activities related to deforestation, the burning of fossil fuels, and the rise of carbon emissions elaborated. For no visible reason, *“the people”* become morally faulty and start to act in an environmentally destructive fashion. As story 2 puts it: “*The people of the world had started to become careless and selfish. They cut down the forests, polluted the air and water, and consumed resources at an alarming rate.”* Thus, the societal dynamics of global warming become blurred. For instance, capitalism, economic growth, or certain modes of production (such as industrial farming) are never part of the stories. In this sense, ChatGPT’s narrative shows similarities to the current debates on global environmental change and the so-called Anthropocene. The term “Anthropocene” refers to “the current epoch in which humans and our societies have become a global geophysical force” (Steffen et al. 2007, p. 614). Although some authors, such as Chakrabarty (2021), regard the concept of the Anthropocene as analytically beneficial because its impacts will outlast economic systems such as capitalism, the dominant discourse on climate change and related discussions concerning the Anthropocene have also been criticized for addressing current socio-ecological crises as universally “man-made.” According to this perspective, the Anthropocene and global warming are not caused by the *Anthropos*, humans as a species, but by specific political and economic drivers (Moore 2016). Therefore, critics see a tendency toward de-politicization in the dominant (re-)presentation of climate change and the Anthropocene with its focus on humanity as a whole (Swyngedouw 2010; Swyngedouw and Ernstson 2018). A similar criticism can be leveled at ChatGPT’s stories of climate change. Not only its solutions for climate change but also ChatGPT’s description of its origin show a universalizing character masking any kind of societal and political differentiation. Thus, ChatGPT’s narrative on climate change reproduces the post-political qualities in current debates on global warming.

### The (non-)issue of climate justice

The absence of questions of power relations or conflicts of interest also becomes manifest in the neglect of climate justice issues in ChatGPT’s narrative. The stories on climate change show that ChatGPT does filter information on climate change, as none of the stories includes evident misinformation. The narrative of the stories is generally in line with recognized knowledge on climate change as presented in the reports produced by the Intergovernmental Panel on Climate Change (IPCC). This holds true for the causes of global warming, its impacts, as well as options for mitigation (see, for instance, the latest IPCC report 2023). A recent study (Bergener et al. 2023), using a different methodology, also found that ChatGPT’s climate-related responses show a high degree of accuracy.

However, the IPCC, as the most authoritative source on the topic, stresses that the impact of climate change differs from region to region (Lee et al. 2023). Additionally, the IPCC reports highlight that some social groups suffer more from the consequences of climate change than others because their situation cannot be adapted to their altered living conditions (Lee et al. 2023). In general, disadvantaged social groups are expected to be more vulnerable to climate impacts such as extreme weather. In the societal and academic discourse on the climate crisis, this is usually addressed by the term “climate justice” (Harlan et al. 2015). Climate movements such as Fridays for Future (FFF) highlight this aspect of the climate crisis (Martiskainen et al. 2020). ChatGPT’s stories on climate change omit this topic of unbalanced risks.

Discussions on climate justice also deal with the question of a society’s fair burden in mitigating climate change, which has been constitutive for the international negotiations on climate change from the very beginning. Already Article 3 of the United Nations Framework Convention on Climate Change (UNFCCC) from 1992 states that “the Parties protect the climate system for the benefit of present and future generations of humankind, on the basis of equity and in accordance with their common but differentiated responsibilities and respective capabilities.” Taking historical emissions into account, responsibility for the climate crisis stems to more than 90% from societies of the Global North (Hickel 2020). “Accordingly, the developed country Parties should take the lead in combating climate change and the adverse effects thereof” (UNFCCC 1992 article 3). Today, per capita carbon emissions are still highest in societies of the Global North such as the USA and most European countries, and within these countries people with a high income emit more than relatively poor groups (Bruckner et al. 2022). However, the dimension of climate justice and, more specifically, the aspect of “differentiated responsibilities” is missing entirely from ChatGPT’s stories on climate change. According to ChatGPT’s narrative it is *“the people of the world”* who *“realized that they had the power to change their ways and repair the damage they had caused”* (story 6).

A further aspect of climate justice *is* represented in ChatGPT’s stories: Due to the escalating dynamic of climate change, young people and future generations are expected to be affected more severely by the impacts of global warming. This is reflected by ChatGPT, as again the quote from story 3 makes clear: “*The people of the world began to realize that they had a responsibility to protect the earth, not just for themselves, but for future generations as well”*.

Previously missing aspects of climate change do appear in our resampled material that ChatGPT generated in answering the prompt “Tell us a story about climate justice.” In particular, the *“disproportionate impact of climate change on vulnerable neighborhoods”* (story 19), or “*vulnerable populations around the world”* (story 16) is addressed. Furthermore, the discrepancy between polluters, persons responsible, and those affected is addressed. For instance, story 17 states: “*The villagers of Harmony Bay were paying the price for the actions of people they had never met”*. Even the issue of just burden sharing is mentioned: *"it’s also about ensuring that the burden of climate change is shared equitably among all nations and generations”* (story 17).

In short, when ChatGPT is explicitly asked about climate justice instead of climate change the most crucial aspects of climate justice are addressed. However, “climate justice” is already a relatively specific concept in the discourse surrounding climate change. It requires particular prior knowledge, which suggests that non-specialist users would less frequently select it as a prompt than a more generic version.

## Conclusion and outlook

Although we did not explicitly ask ChatGPT for a fictional story, the AI locates all its stories about the factual climate change of our time in a fictional setting. This seems to be significant in two ways: First, people do use ChatGPT to generate fictional plots. According to media reporting, it is even used to write bedtime stories for children (Holmes 2023). Such stories influence people’s attitudes, and therefore, the kind of narrative ChatGPT produces on global warming is highly relevant. Second, in recent years, voices calling for more storytelling and narratives in climate change communication have grown louder, since these methods are perceived as being capable of reducing the action gap in climate change mitigations (Harris 2020). According to research, storytelling and narratives might help “audiences understand and relate to the information” (Bloomfield and Manktelow 2021, p. 311). Consequently, climate change information embedded in fictional narratives becomes especially relevant.

ChatGPT’s narrative on climate change strongly resembles the dominant discourse on global warming as presented by political agents, international organizations, and prestige media: Climate change is caused by carbon emissions from burning fossil fuels and deforestation, and global warming is an immense threat with disastrous consequences for humanity. Not a single story generated by ChatGPT conveyed elements of climate denialism (though some stories included passages on people denying climate change). By putting forward ideas of ecological modernization, ChatGPT’s narrative is close to the current discourse that focuses mostly on technological solutions for the climate crisis (Sommer and Welzer 2014; Hickel 2021).

ChatGPT furthermore reproduces tendencies that attribute the climate crisis and further ecological crises to humans as a species and neglect socially differentiated analyses of their drivers. This pattern is particularly prominent in discourses that are coined by natural scientists, such as the discussion on the Anthropocene. Similarly, ChatGPT does not differentiate adequately between social groups in regard to their economic, political, and technological capacities; neither in respect to their vulnerability to climate impacts, nor regarding their responsibility for causing the climate crisis. Of course, stories of ca. 380 words are necessarily rather abstract and cannot cover every aspect of the climate crisis. However, it is telling that none of ChatGPT’s stories on climate change addressed the issue of climate justice beyond intergenerational justice. Only if ChatGPT is explicitly asked to tell a story about climate justice does it integrate relevant aspects into its general narrative, which remains otherwise largely unchanged.

The similarities between ChatGPT’s narrative and the dominant public discourse raise a number of questions that might trigger further research. For instance, is ChatGPT’s narrative always as limited as the dominant societal imaginary on climate change? Our analysis already highlighted that more radical approaches to tackling the climate crisis and reaching ecological sustainably (such as *degrowth*) are missing from ChatGPT’s stories. Control imaginaries (Adloff and Neckel 2019) are also not part of the ChatGPT narrative on climate change; they only appear if ChatGPT is asked about climate justice.

Which further alternative discourses are not represented? What is the situation regarding proposals from the Global South relating to the global environmental crises such as *Buen Vivir* (Acosta and Abarca 2018)? Postcolonial perspectives on climate change (Ghosh 2021), for instance, are also missing. Is ChatGPT mainly trained on the Western discourse on climate change?

An analysis of AI-generated texts employing qualitative social research methodology has clear limitations: Reconstructing ChatGPT’s narrative on climate change does not allow conclusions to be drawn on the question why and how ChatGPT is writing this specific story. OpenAI’s usage policies prohibit the use of its services to generate “hateful, harassing, or violent content.” Additionally, the use of OpenAI software for “fraudulent or deceptive activity, including […] disinformation” is disallowed (OpenAI: Usage Policies 2023). ChatGPT uses filters in order to prevent the generation of content that violates these policies. The issue of anthropogenic climate change is not directly addressed by the company’s usage policies. However, the creation of a highly uniform narrative that is grosso modo in line with the science on climate change suggests that corresponding filters do exist. This aligns with quantitative analyses showing that “ChatGPT seems to hold a bias towards progressive views” (Rutinowski et al. 2023, p.1).

The specific formulation of the stimulus, i.e. the prompt, strongly influences the output of AI chatbots. For our explorative study, we decided to use a very generic prompt. However, pretests with alternative prompts such as “climate crisis” or “climate catastrophe” showed that ChatGPT then depicts the impacts of global warming as far more severe. For instance, none of the stories with the stimulus “climate catastrophe” had a happy end. Our analysis of stories on “climate justice” showed that aspects of this concept were introduced, although the rough narrative of the stories remained the same. Furthermore, control samples of highly specific prompts (such as “Tell me about climate justice. What does it mean and what does the concept entail?”) also generate far more specific answers. Asking ChatGPT more precisely about aspects that are missing in its general narrative on climate change was not possible in our limited, explorative analysis. This could be the subject of a more comprehensive and systematic study. Additionally, our findings highlighted that the issue of prompt engineering is of vital importance. Finally, since ChatGPT is continuously being developed further, it will also be interesting to see how its narrative on climate change alters over time. Interdisciplinary research on the AI–society nexus has just begun.

### Supplementary Information

Below is the link to the electronic supplementary material.

Supplementary file1 (PDF 480 KB)
