# Supplementary material for: “In the end, the story of climate change was one of hope and redemption”: ChatGPT’s narrative on global warming
Source: Ambio. 2024 Mar 2;53(7):951–9. doi: 10.1007/s13280-024-01997-7 (PMC11101383; doi:10.1007/s13280-024-01997-7)
Supplement: Supplementary file 1 — Supplementary file1 (PDF 480 KB) [file 13280_2024_1997_MOESM1_ESM.pdf]

Ambio

Supplementary Information
This supplementary information has not been peer reviewed.

Title: "In the end, the story of climate change was one of hope and redemption." ChatGPT's Narrative on Global Warming

Appendix 1

Codesystem: „Tell us a story about climate change"

(ChatGPT Version 3.5, Data collection March 2023)

List of Codes	Frequency
**Initial Situation**	
Paradisiac Place	3
Healthy Planet	13
**Causes for Climate Change (Human Destruction)**	
Caused by Human Activity (Overexploitation)	16
Consumption	1
Driving	3
Exploitation of Resources	5
Pollution	4
GHG/Carbon Emission	17
Fossil Fuel	11
Deforestation	9
**Knowledge about Causes**	
Science // Listen to the Science	7
Old Wise Man	1
Climate Change Impacts	3

Appendix 1

**Oceans and Cryosphere**	
Oceans Heat Up	2
Rising Sea	10
Melting Caps	7
Season Changes	
Cold Winter	2
Hot Summer	5
Mild Winter	3
Seasons Unstable	3
Extreme Weather (Events)	10
Natural Disasters	3
Droughts/Bad Harvest	15
Heatwaves	4
Rising Temperatures	3
Hurricanes/Storms	10
Flood	4
Fire	2
**Impacts on Environment**	
Water Pollution	1
Threat to Ecosystems	1
Waldsterben	1
Extinction of Species	7

Air Quality	5
Climate Change as a Threat	7
Planet Irreparably Damaged	1
Societal Impacts: Loss and Damage	12
**First Reactions on Climate Change**	
Helplessness	5
Scepticism/Ignoring/Denial	9
Shock	1
Adaptation not Possible	1
**Climate Action (Mitigation)**	
**Views, Values and Knowledge**	
Moral Obligation	1
People Part of Nature	2
Confidence/Satisfaction With Own Activism	2
**Modes of Action**	
Governmental Action	9
Collaborative Action	3
Activism	4
Protest	1
Raising Awareness	4
Social Media	1
Address Governments	2

Appendix 1

**Actors**	
NGOs	1
Companies	2
Governments, Political Leaders	15
**Individuals**	
Knowledge	1
Change is Possible by Action	11
**Motives & Faciliators**	
Cooperation	16
Responsibility	4
Generational Justice	12
Huge Efforts	15
Don't Give Up	
**Measures**	2
Behavior Change Necessary	15
Protect Environment	2
Individual Changes	2
Reduce Consumption	4
Driving	2
Carbon Footprint	5
Carbon Footprint	5
(Plastic) Waste Reduction/Recycling	4

New Technology	2
Renewable Energy	13
Restoring Ecosystems	3
Conserving Resources	1
Aforestation	7
**Outcomes/Endings**	
Hope	4
Healing	14
Lesson Learned	7
Climate Change as an Ongoing Challenge	11
Indifferent Ending	1
Inconsistencies within Stories	4

Appendix 2

Table 1: Storyline Matrix

<table>
<tr><td>Story 1</td><td colspan="2">Story 2</td><td>Story 3</td><td>Story 4</td><td>Story 5</td></tr>
<tr><td>Healthy Planet</td><td colspan="2">Healthy Planet</td><td>Healthy Planet</td><td>Like our world</td><td>Healthy Planet</td></tr>
<tr><td>Humans Overexploit</td><td colspan="2">Humans Overexploit</td><td>Fist Signs and Impacts</td><td>Fist Signs and Impacts I denial</td><td>Fist Signs and Impacts</td></tr>
<tr><td>Fist Signs and Impacts</td><td colspan="2">Fist Signs and Impacts</td><td>Threat</td><td>Threat</td><td>Threat</td></tr>
<tr><td>Threat</td><td>Threat</td><td rowspan="2">Simul-taneous</td><td>Humans Overexploit</td><td>Humans Overexploit</td><td>Humans Overexploit</td></tr>
<tr><td>Hope I Mitigation</td><td>Mitigation</td><td>Mitigation</td><td>Mitigation</td><td>Mitigation</td></tr>
<tr><td>Lesson Learned / Ongoing Challenge</td><td colspan="2">Hope & Healing</td><td>Hope & Healing</td><td>Hope & Healing</td><td>Hope & Healing</td></tr>
<tr><td>Hope & Healing</td><td colspan="2">Lesson Learned / Ongoing Challenge</td><td>Lesson Learned / Ongoing Challenge</td><td>Lesson Learned / Ongoing Challenge</td><td>Lesson Learned / Ongoing Challenge</td></tr>
</table>

<table>
<tr><td>Story 6</td><td colspan="2">Story 7</td><td>Story 8</td><td>Story 9</td><td>Story 10</td></tr>
<tr><td>Like our world</td><td colspan="2">Healthy Planet</td><td>Threat (n year 2050)</td><td>Healthy Planet</td><td>Healthy Planet</td></tr>
<tr><td>Fist Signs and Impacts</td><td colspan="2">Fist Signs and Impacts</td><td>Healthy in the past</td><td>Fist Signs and Impacts</td><td>Humans Overexploit</td></tr>
<tr><td>Humans Overexploit</td><td colspan="2">Humans Overexploit</td><td>Mitigation</td><td>Ignorance I Threat</td><td>Fist Signs and Impacts</td></tr>
<tr><td>Skepticism</td><td>Threat</td><td rowspan="2">Simul-taneous</td><td>Skepticism I Ignorance</td><td>Humans Overexploit</td><td>Threat</td></tr>
<tr><td>Mitigation (?)</td><td>Mitigation</td><td>Mitigation</td><td>Mitigation</td><td>Mitigation</td></tr>
<tr><td>Threat</td><td colspan="2">Hope & Healing</td><td>Hope & Healing</td><td>Hope & Healing</td><td>Hope & Healing</td></tr>
<tr><td>Indifferent Ending</td><td colspan="2">Lesson Learned / Ongoing Challenge</td><td>Lesson Learned / Ongoing Challenge</td><td>Lesson Learned / Ongoing Challenge</td><td>Lesson Learned / Ongoing Challenge</td></tr>
</table>

<table>
<tr><td>Story 11</td><td>Story 12</td><td colspan="2">Story 13</td><td>Story 14</td></tr>
<tr><td>Healthy Planet</td><td>Like Our World</td><td colspan="2">Healthy Planet</td><td>Healthy Planet</td></tr>
<tr><td>Humans Overexploit</td><td>Humans Overexploit</td><td colspan="2">Human Destroy</td><td>Humans Overexploit</td></tr>
<tr><td>Fist Signs and Impacts</td><td>Fist Signs and Impacts</td><td colspan="2">Fist Signs and Impacts</td><td>Fist Signs and Impacts</td></tr>
<tr><td>Ignorance I Threat</td><td>Threat</td><td>Threat</td><td rowspan="2">Simul-taneous</td><td>Ignorance I Threat</td></tr>
<tr><td>Mitigation</td><td>Mitigation I ignorance</td><td>Mitigation</td><td>Mitigation</td></tr>
<tr><td>Hope & Healing</td><td>Hope & Healing</td><td colspan="2">Lesson Learned / Ongoing Challenge</td><td>Hope & Healing</td></tr>
<tr><td>Lesson Learned / Ongoing Challenge</td><td>Lesson Learned / Ongoing Challenge</td><td colspan="2">Hope & Healing</td><td>Lesson Learned / Ongoing Challenge</td></tr>
</table>

Appendix 3

Codesystem: “Tell us a story about climate justice”

(ChatGPT Version 3.5, Data collection of November 2023)

List of Codes	Frequency
**Initial Situation**	
"Not-So-Distant Future"	2
Paradisiac Place	1
Healthy Planet	3
**Causes for Climate Change (Human Destruction)**	
Caused by Human Activity (Overexploitation)	1
Faraway Industries	1
GHG/Carbon Emission	1
Knowledge about Causes	1
Science // Listen to the Science	2
**Climate Change Impacts**	
Oceans and Cryosphere	
Oceans Heat Up	1
Rising Sea	4
Melting Caps	1
Extreme Weather (Events)	2
Unpredictable Weather	1
Droughts/Bad Harvest (+)	1
Heatwaves	2
Hurricanes/Storms	2

Appendix 3

Flood	2
Impacts on Environment	
Dying Coral Reefs	1
Threat to Ecosystems	2
Extinction of Species	1
Climate Change as a Threat	1
Societal Impacts: Loss and Damage	4
Failing/Collapsing Industry	2
Hunger	1
Disproportionately Affected Vulnerable Populations (in vivo)	5
Wealthy Elite Shielded from Effects	1
First Reactions on Climate Change	
Anger	1
Sadness	1
Scepticism/Ignoring/Denial	1
**Climate Action (Mitigation)**	1
Adaptation	1
Building Sea Walls	1
Views, Values and Knowledge	
Moral Obligation	2
People Part of Nature	2
Modes of Action	
Donating	1
Governmental Action	2
Village Elders	1

Appendix 3

Collaborative Action	10
Climate Justice Council	2
Diversity	3
Activism	11
Solidarity	2
Activist Girl (Maya)	4
Protest	4
Raising Awareness	5
Art	1
Address Governments	4
Actors	
Companies	4
Governments, Political Leaders (also regional)	4
World Leaders	1
Motives & Faciliators	
Climate Justice	10
Representation of Minorities/Small Communities	1
Unequal Distribution of Consequences	8
Cooperation	2
Responsibility	4
Generational Justice	2
Measures	
Changing Attitudes	2
Helping the Vulnerable	1
Regulations, Policies	5

Sustainable Practices	5
Protecting Coastlines	1
Work	
Job Retaining Programs	2
Behavior Change Necessary	
Community Garden	1
Transportation	1
Individual Changes	1
Education	6
Reduce Consumption	2
Carbon Footprint	4
(Plastic) Waste Reduction/Recycling	1
New Technology	1
Renewable Energy	4
Restoring Ecosystems	1
Conserving Resources	1
Aforestation	1
**Outcomes/Endings**	
Role Model	10
Sustainibility Achievable	2
Spillover	4
Everyone can make a Difference	4
Hope	4
Healing	3
Lesson Learned	1

Appendix 3

Climate Change as an Ongoing Challenge	2
Indifferent Ending	1
